# New insights into genetic susceptibility of COVID-19: an *ACE2* and *TMPRSS2* polymorphism analysis

**DOI:** 10.1186/s12916-020-01673-z

**Published:** 2020-07-15

**Authors:** Yuan Hou, Junfei Zhao, William Martin, Asha Kallianpur, Mina K. Chung, Lara Jehi, Nima Sharifi, Serpil Erzurum, Charis Eng, Feixiong Cheng

**Affiliations:** 1 Genomic Medicine Institute, Lerner Research Institute, Cleveland Clinic, Cleveland, OH 44195 USA; 2 Department of Systems Biology and Department of Biomedical Informatics, Herbert Irving Comprehensive Center, Columbia University, New York, NY 10032 USA; 3 Department of Molecular Medicine, Cleveland Clinic Lerner College of Medicine, Case Western Reserve University, Cleveland, OH 44195 USA; 4 Department of Cardiovascular Medicine, Heart, Vascular and Thoracic Institute, Cleveland Clinic, Cleveland, OH 44195 USA; 5 Lerner Research Institute, Cleveland Clinic, Cleveland, OH 44195 USA; 6 Department of Genetics and Genome Sciences, School of Medicine, Case Western Reserve University, Cleveland, OH 44106 USA; 7 Case Comprehensive Cancer Center, School of Medicine, Case Western Reserve University, Cleveland, OH 44106 USA

**Keywords:** Angiotensin-converting enzyme 2 (ACE2), Coronavirus, COVID-19, Genetic susceptibility, SARS-CoV-2, *TMPRSS2*

## Abstract

**Background:**

Coronavirus Disease 2019 (COVID-19), caused by the severe acute respiratory syndrome coronavirus 2 (SARS-CoV-2), has now been confirmed worldwide. Yet, COVID-19 is strangely and tragically selective. Morbidity and mortality due to COVID19 rise dramatically with age and co-existing health conditions, including cancer and cardiovascular diseases. Human genetic factors may contribute to the extremely high transmissibility of SARS-CoV-2 and to the relentlessly progressive disease observed in a small but significant proportion of infected individuals, but these factors are largely unknown.

**Main body:**

In this study, we investigated genetic susceptibility to COVID-19 by examining DNA polymorphisms in *ACE2* and *TMPRSS2* (two key host factors of SARS-CoV-2) from ~ 81,000 human genomes. We found unique genetic susceptibility across different populations in *ACE2* and *TMPRSS2.* Specifically, *ACE2* polymorphisms were found to be associated with cardiovascular and pulmonary conditions by altering the angiotensinogen-ACE2 interactions, such as p.Arg514Gly in the African/African-American population. Unique but prevalent polymorphisms (including p.Val160Met (rs12329760), an expression quantitative trait locus (eQTL)) in *TMPRSS2*, offer potential explanations for differential genetic susceptibility to COVID-19 as well as for risk factors, including those with cancer and the high-risk group of male patients. We further discussed that polymorphisms in *ACE2* or *TMPRSS2* could guide effective treatments (i.e., hydroxychloroquine and camostat) for COVID-19.

**Conclusion:**

This study suggested that *ACE2* or *TMPRSS2* DNA polymorphisms were likely associated with genetic susceptibility of COVID-19, which calls for a human genetics initiative for fighting the COVID-19 pandemic.

## Background

Coronaviruses (CoVs), so named for their “crown-like” appearance by electron microscopy, are a large family of viruses that spread from animal hosts (e.g., bats, civet, cats) to humans, causing life-threatening respiratory diseases like Middle East respiratory syndrome (MERS) and severe acute respiratory syndrome (SARS) [1]. As of June 18, 2020, over 8.4 million cases and 450,000 deaths resulting from infection by a novel SARS coronavirus, SARS-CoV-2 (also termed Coronavirus Disease 2019 or COVID-19), have now been confirmed worldwide; furthermore, there have been more than 2.2 million confirmed cases and over 110,000 deaths due to the COVID-19 pandemic in the USA alone [2]. Unlike other CoVs, SARS-CoV-2 has had much larger global spread and has impacted more people than SARS-CoV-1 and MERS-CoV combined [1]. Morbidity and mortality due to COVID-19 rise dramatically with age and co-existing health conditions, including cancer and cardiovascular diseases, and while most infected individuals recover, even very young and otherwise healthy patients may unpredictably succumb to this disease [3]. These observations beg the question of how much of the variation in COVID-19 disease severity may be explained by genetic susceptibility. Human genetic factors may contribute to the extremely high transmissibility of SARS-CoV-2 and to the relentlessly progressive disease observed in a small but significant proportion of infected individuals; yet, these factors are largely unknown. Development of new preventive and/or therapeutic strategies for COVID-19 will be greatly facilitated by systematic identification of host genetic pathways and DNA polymorphisms (variants) which modulate the risk of infection and severe illness, including the overexuberant immune response to the virus that often portends a poor outcome.

**Fig. 1:**
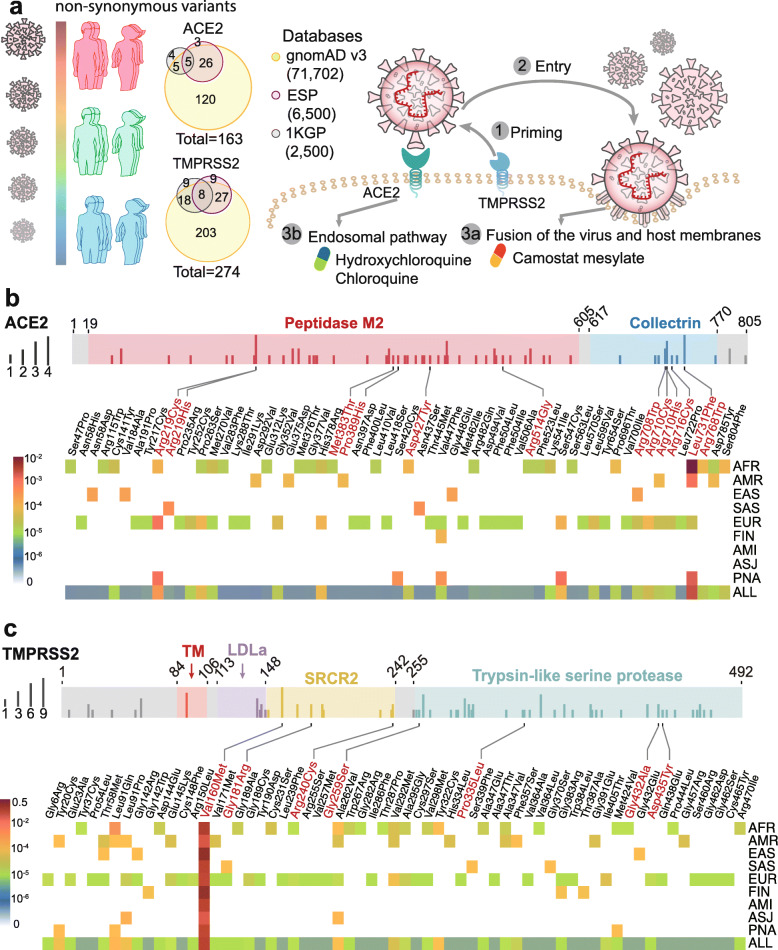
The coding-region variants in *ACE2* and *TMPRSS2* from ~ 81,000 human genomes across 8 populations. **a** Coding-region variants in the genes encoding angiotensin-converting enzyme 2 (*ACE2*) and transmembrane protease serine 2 (*TMPRSS2*) across three human genome databases: (i) Genome Aggregation Database (gnomAD v3), (ii) Exome Sequencing Project (ESP), and (iii) 1000 Genomes Project (1KGP). SARS-CoV-2 utilizes the host cell factors angiotensin-converting enzyme 2 (ACE2) for entry into cells and the host transmembrane serine protease TMPRSS2 for SARS-CoV-2 spike (S) protein priming, offering potential pathway for therapeutic development in treatment of COVID-19. **b** Distribution of 61 deleterious variants in the ACE2 coding region identified in gnomAD (v3). Polyphen2 > 0.96 and CADD scores > 20 as cutoff identify putative deleterious variants. The upper panel using 3 colors shows the functional domains of ACE2, and the height of the vertical line represents the number of populations that carry this variant. The lower heatmap shows the allele frequencies (color key) of a variant across different populations. **c** Distributions of 63 putative deleterious variants in the *TMPRSS2* coding region using the same approach of **b**. AFR, African/African-American; AMI, Amish; AMR, Latino/Admixed American; ASJ, Ashkenazi Jewish; EAS, East Asian; FIN, Finnish; EUR, Non-Finnish European; SAS, South Asian; PNA, population not assigned

Not only has the COVID-19 pandemic had huge health and economic impacts in 188 countries/regions across the world, but the disease has also struck in different racial/ethnic subpopulations. Large genetic studies in populations of geographically diverse ancestry have demonstrated substantial genetic variation in protein-coding regions, with widely varying allele frequencies [4]. SARS-CoV-2 infection depends on the host cell factors angiotensin-converting enzyme 2 (ACE2) for entry into cells and the host transmembrane serine protease TMPRSS2 for SARS-CoV-2 spike (S) protein priming [5] (Fig. 1a). ACE2, encoded on the X-chromosome, catalyzes the conversion of angiotensin II to angiotensin-(1–7), which acts as a vasodilator and exerts important modulatory effects on the cardiovascular system. *TMPRSS2* is a key gene in prostate cancer, as an associated translocation drives ETS-family oncogene expression in a large proportion of tumors [6]. The distribution of *ACE2* expression has recently been investigated by single-cell RNA sequencing, and the expression of both *ACE2* and *TMPRSS2* are likely to dictate SARS-CoV-2 tissue tropism [7]. Clinical studies have reported that incidence and mortality rates are significantly different between male and female COVID-19 patients, and the disease is associated with pre-existing conditions, such as cancer and cardiovascular disorders, in particular individuals with hypertension receiving anti-hypertensive medications [8]. Therefore, a systematic investigation of the functional polymorphisms in *ACE2* and *TMPRSS2* among different populations could pave the way for precision medicine and personalized treatment strategies for COVID-19.

## *ACE2* polymorphism analysis across different populations

Here, we investigated genetic susceptibility to COVID-19 by examining DNA polymorphisms in *ACE2* (OMIM 300335) and *TMPRSS2* (OMIM 602060) genes. We assembled a total of 437 non-synonymous single-nucleotide variants (SNVs) in the protein-coding regions of *ACE2* and *TMPRSS2* (Fig. 1a) from three databases: (i) Genome Aggregation Database (gnomAD v3: gnomad.broadinstitute.org, covering 9 geographical areas), (ii) Exome Sequencing Project (ESP: evs.gs.washington.edu/EVS/), and (iii) 1000 Genomes Project (1KGP, www.internationalgenome.org). We used ANNOVAR [9] to annotate all non-synonymous variants. By applying Polyphen2 and CADD (Combined Annotation Dependent Depletion) scores, we identified 63 potentially deleterious variants in *ACE2* (61 in gnomAD) and 68 deleterious variants in *TMPRSS2* (63 in gnomAD).

**Fig. 2:**
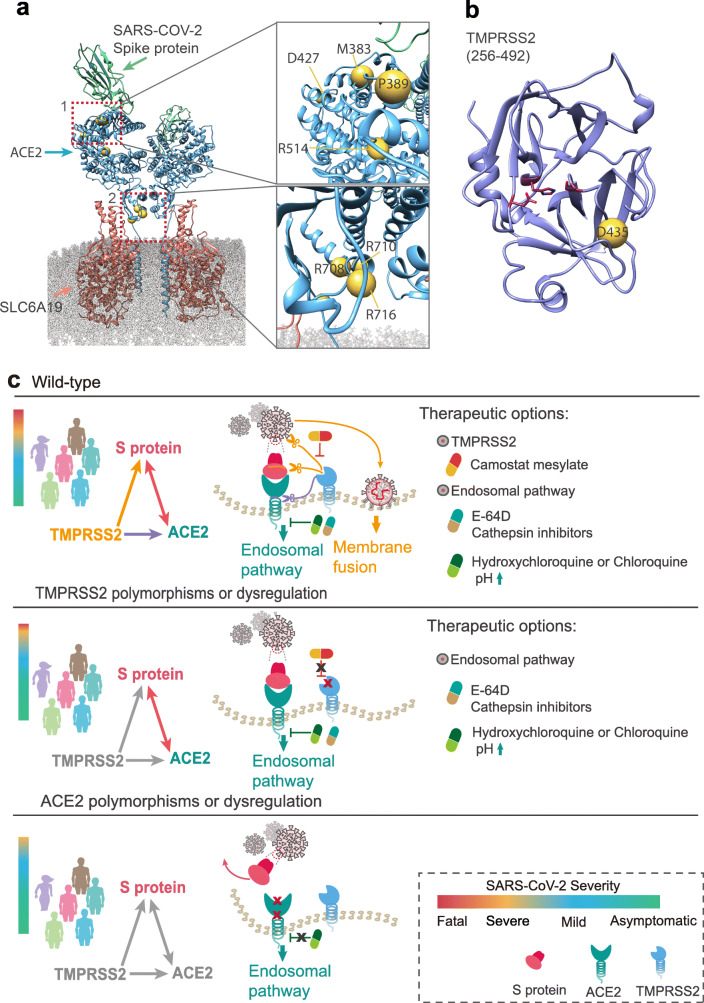
Structural view of the coding-region variants in *ACE2* and *TMPRSS2* and a proposed pharmacogenomics model of effective combination therapies for COVID-19. **a** Full-length structures of the sodium-dependent neutral amino acid transporter B(0)AT1 (SLC6A19, red)–ACE2 (blue) heterodimer in its homodimeric form complexed with the receptor binding domain (RBD, mint) of SARS-CoV-2 (PDB ID: 6M17). Highly deleterious variants are labeled as yellow spheres on ACE2. Insets depict mutations in residues 383 through 427 (top) and residues 708 through 731 (bottom). **b** Homology model of the catalytic chain (256–492) of TMPRSS2. Highly deleterious mutations are labeled as yellow spheres. **c** A proposed model of effective combination therapies (i.e., hydroxychloroquine, E-64D (a protease inhibitor), and camostat mesylate (an approved TMPRSS2 for treatment of chronic pancreatitis in Japan)) for COVID-19 by blocking ACE2 and TMPRSS2 across different populations with three genotypes. Relationship among spike (S) protein of SARS-CoV-2, ACE2, and TMPRSS2 were shown as a triangle, with each pair connecting by physical binding (double-headed arrow) or cleavage (single-headed arrow). We propose three hypotheses for COVID-19 therapeutic options: (i) for patients with wild-type or naïve expression of *ACE2* and *TMPRSS2*, hydroxychloroquine (or chloroquine, or E-64D) combined with camostat may offer more clinical benefit; (ii) for patients with polymorphisms or dysregulation on *TMPRSS2*, hydroxychloroquine or chloroquine monotherapy may offer more clinical benefit; and (iii) for patients with polymorphisms or dysregulation on *ACE2*, the patients who might have mild symptoms can recover in a short period. All three pharmacogenomics models for COVID-19 must be validated both experimentally and clinically before being used in patients

We found that the distribution of deleterious variants in *ACE2* differs among 9 populations in gnomAD (v3). Specifically, 39% (24/61) and 54% (33/61) of deleterious variants in *ACE2* occur in African/African-American (AFR) and Non-Finnish European (EUR) populations, respectively (Fig. 1b). Prevalence of deleterious variants among Latino/Admixed American (AMR), East Asian (EAS), Finnish (FIN), and South Asian (SAS) populations is 2–10%, while Amish (AMI) and Ashkenazi Jewish (ASJ) populations do not appear to carry such variants in *ACE2* coding regions (Fig. 1b). Specifically, several variants, including p.Met383Thr, p.Pro389His, and p.Asp427Tyr, have been reported to slightly inhibit the interaction between ACE2 and the spike protein of SARS-CoV-1 [10], which caused the first global SARS-CoV-1 outbreak. Only AFR populations carry p.Met383Thr and p.Asp427Tyr variants, with allele frequencies of 0.003% and 0.01%, respectively (Fig. 1b). The p.Pro389His only occurs in the AMR populations, with an allele frequency of 0.015%. The p.Arg514Gly is a low allele frequency (0.003%) variant in AFR populations and is also somatically mutated in colon cancers and melanomas from The Cancer Genome Atlas (TCGA: https://portal.gdc.cancer.gov). This *ACE2* variant is located in the angiotensinogen (AGT)-ACE2 interaction surface, which is anticipated to influence the renin-angiotensin system (RAS) function. The RAS is critical for regulation of blood pressure, sodium, and fluid balance, and its dysfunction is associated with cardiovascular and kidney disorders [11]. Residues Arg708/710/716 are located in the dimeric interface of ACE2 (Fig. 2a), and they are essential for its cleavage by TMPRSS2; this processing is required for augmentation of SARS-S-driven entry into host cells [12]. The EUR population carries the p.Arg708Trp, p.Arg710Cys, p. Arg710His, and p.Arg716Cys variants with allele frequency of 0.01~0.006% (Fig. 1a), while the EAS and the AMR populations only carry p.Arg708Trp and p.Arg710His with allele frequency of 0.04% and 0.01% respectively. In addition to these four variants, p.Leu731Phe has the highest allele frequency in the AFR and EUR populations. We further inspected the expression quantitative trait loci (eQTL) for *ACE2* using the GTEx [13] and QTLbase [14] databases. We did not find any eQTLs for *ACE2* from the GTEx, while we found one weak eQTL associated with *ACE2* non-synonymous SNP (rs41303171) in the kidney from the QTLbase [14].

Altogether, these comparative genetic analyses suggest that *ACE2* genomic variants may play important roles in susceptibilities to COVID-19 and its associated cardiovascular conditions by altering AGT-ACE2 pathway (i.e., p.Arg514Gly). In addition to differential polymorphisms which may explain susceptibility and even outcome in different ethnic populations, the fact that *ACE2* is localized to Xp22.2 may help explain the observed male-associated risk. As such, even in the absence of variation in this gene, the monoallelic versus biallelic presence of this gene may impact the natural history and prognosis of COVID-19 in males.

## *TMPRSS2* polymorphism analysis across different populations

TMPRSS2 enzyme activity is important for coronavirus spread and pathogenesis in the infected host [15]. Our analysis indicates 4% (11/274) of non-synonymous variants of TMPRSS2 are stop-gained mutations and carried by AFR and EUR with low allele frequency (7.0 × 10^−6^~1.4 × 10^−5^). Meanwhile, 35% (22/63) and 59% (37/63) of deleterious variants in TMPRSS2 coding regions are carried by the AFR and EUR populations from gnomAD (v3), respectively (Fig. 1c). Each of the EAS, SAS, and FIN populations only carries 4 deleterious variants. We found 6 germline deleterious variants (p.Val160Met, p.Gly181Arg, p.Arg240Cys, p.Gly259Ser, p.Pro335Leu, and p.Gly432Ala) in the *TMPRSS2* coding region, which are also identified as somatic mutations occurring in different cancer types from TCGA and COSMIC databases (https://cancer.sanger.ac.uk/cosmic).

We further evaluated the eQTL profile of *TMPRSS2* using the GTEx [13] and QTLbase databases [14] as well. We found two eQTLs associated with *TMPRSS2* non-synonymous SNPs (rs12329760 (encoding p.Val160Met), *p* = 4.54 × 10^−5^; rs75603675, *p* = 0.009) in the kidney and bone, respectively, using the QTLbase database [14], while there are no known eQTLs associated with *TMPRSS2* non-synonymous SNPs from GTEx [13]. Notably, all populations carry p.Val160Met variants with the highest allele frequency (~ 25%), especially for the EAS population at a 40% allele frequency. Asp435 is a key residue for catalytic substrate binding of TMPRSS2 (Fig. 2b). We found that the p.Asp435Tyr, which has low allele frequency, is carried by the EUR population only (Fig. 1c). These unique but prevalent polymorphisms in *TMPRSS2* offer potential explanations for differential genetic susceptibility to COVID-19 as well as for risk factors, including those with cancer and the high-risk group of male patients. Because *TMPRSS2* is located on 21q22.3, we could speculate that individuals with Down syndrome would be at high risk for COVID-19 infection. In addition, oncogenic roles of *TMPRSS2* may be linked to poor outcomes with COVID-19 as well [16], which should be studied in the future. Using single-cell RNA-sequencing analysis, Schuler et al. showed that *TMPRSS2* expression was highest in ciliated cells and type I alveolar epithelial cells (AT1) and increased with aging in humans and mice [17]. This observation suggests that developmental regulation of *TMPRSS2* may link the relative protection of infants and children from COVID-19. Thus, it should be of great interest to investigate the age-related polymorphisms for *TMPRSS2*, such as using the Genetic Epidemiology Research on Adult Health and Aging (GERA) cohort [18], in the future.

## Host genetic factors guide personalized treatment of COVID-19

There are currently no approved effective medications against COVID-19. Several national and international research groups are working on the development of vaccines to prevent COVID-19, but effective vaccines not likely to be available for many months. Several potentially repurposable drugs (Fig. 2c), including melatonin [19], hydroxychloroquine, and chloroquine, are under investigation for treatment of COVID-19 [20]. A primary mechanism-of-action of hydroxychloroquine and chloroquine is to inhibit virus entry by targeting the endosomal pathway [20]. Hydroxychloroquine and chloroquine is known to increase the pH of endosomes, which inhibits membrane fusion, a required mechanism for viral entry into the cell [21]. Additionally, inhibition of SARS-CoV-2 could be due to differential glycosylation of both ACE2 and the spike protein [21]. As shown in Fig. 1b, several variants identified in the AFR and AMR populations, including p.Met383Thr, p.Pro389His, and p.Asp427Tyr (the pathogenic variants in ACE2 slightly inhibit interaction with the S protein), may influence the clinical efficacy of hydroxychloroquine or chloroquine. This may help explain why treatment of hydroxychloroquine was not significantly associated with difference in in-hospital mortality [22]. However, further pharmacogenomic studies that integrate drug response and genetic data from patients with COVID-19 are urgently needed.

In addition to the endosomal pathway, fusion of viral and host cellular membranes through S protein conformational changes is another way for coronavirus entry into the host cell [23]. This process can be blocked by a TMPRSS2 inhibitor (camostat mesylate, a drug approved in Japan) [5]. The mechanisms whereby TMPRSS2 promotes cellular entry of SARS-CoV-2 can be summarized by two aspects based on its proteolytic function (Fig. 2). The first is S protein cleavage at S1/S2 and S2’ sites, which might be the reason why SARS-CoV-2 entry into cells depends on TMPRSS2. The infection and pathogenesis of SARS-CoV-2 depends on the presence of TMPRSS2, in the face of the cellular elevated pH environment [5, 24, 25]. The inhibitors of endosomal acidification such as CatB/L inhibitor E-64D and hydroxychloroquine/chloroquine may only work for TMPRSS2-absence patients who are infected by SARS-CoV-2, and may have less effect or no effect for the patients with wild-type of TMPRSS2 [5, 24]. Therefore, the EUR and AFR populations might be more sensitive to hydroxychloroquine or chloroquine by carrying missense variants and stop-gained variants on *TMPRSS2* (Figs. 1c and 2c). Yet, for patients who have wild-type of *ACE2* and *TMPRSS2*, a combination of camostat with hydroxychloroquine or chloroquine may have better clinical benefit. However, all discussed treatment strategies must be validated by randomized controlled trials before clinical use. The second mechanism is cleavage of ACE2 by TMPRSS2 at Arginine 697 to 716 [12], which enhances viral uptake. Thus, the EUR population with p.Arg708Trp, p.Arg710Cys, p.Arg710His, and p.Arg716Cys variants in ACE2 may have mild symptoms after SARS-CoV-2 infection as ACE2 loses the cleavage site by TMPRSS2 and changes the ACE2 dimer formation [26] (Fig. 2c).

## Discussion and future directions: call for host genetics initiative for COVID-19

A few limitations merit consideration. Current analysis examined massive genomic data from general population, not COVID-19 patient-specific populations. All genetic associations identified in current study are urgently needed to be tested in COVID-19 patients in the near future. As the high-resolution protein structure of TMPRSS2 is not yet available, further functional observations and clinical validation are warranted for all abovementioned genetic and pharmacogenomics findings. We anticipate that large-scale genome-wide association studies (GWAS) are urgently needed to identify likely causal host genetic risk factors for severe COVID-19 outcomes using genetic data from patients with COVID-19; such knowledge will improve risk stratification of individuals exposed to or testing positive for SARS-CoV-2 and allow for precision medicine interventions for COVID-19. A COVID-19 host genetics initiative is already underway to bring together the human genetics research community to generate, share, and analyze data in a search for the genetic determinants of COVID-19 susceptibility, severity, and outcomes [27]. The first COVID-19 GWAS identified the 3p21.31 gene cluster (including SLC6A20, LZTFL1, CCR9, FYCO1, CXCR6, and XCR1) as a genetic susceptibility locus in severe patients with COVID-19 and respiratory failure [28]. Yet, our study aims to look for SNPs associated with disease severity of COVID-19, but not disease susceptibility. In summary, systematic identification of the genetic determinants of COVID-19 susceptibility, severity, and clinical outcome, including both virus and host factors (e.g., *ACE2* and *TMPRSS2* polymorphisms), could guide personalized treatment in the emerging COVID-19 pandemic and even explain current epidemiologic observations (i.e., males, elderly at high risk, and clinical comorbidities) and natural history.

## Conclusions

This comprehensive comparative genetic analysis of approximately 81,000 human genomes suggested possible associations of *ACE2* and *TMPRSS2* DNA polymorphisms with COVID-19 susceptibility, severity, and clinical outcomes. We found that *ACE2* polymorphisms were more likely to be associated with cardiovascular and pulmonary conditions by altering the angiotensinogen-ACE2 interactions, such as p.Arg514Gly in the African/African-American population. Unique but prevalent polymorphisms in *TMPRSS2*, including p.Val160Met (rs12329760), may provide potential explanations for differential genetic susceptibility to COVID-19 as well as for risk factors, including cancer and the high-risk group of male patients. We highlighted that polymorphisms in *ACE2* or *TMPRSS2* could guide personalized treatments (i.e., hydroxychloroquine and camostat) for COVID-19. In summary, this study suggested that *ACE2* or *TMPRSS2* DNA polymorphisms were likely associated with genetic susceptibility to COVID-19, which calls for a human genetics initiative for fighting the COVID-19 pandemic.

## Data Availability

All population genetic data used in this study are free and available at three databases: (i) Genome Aggregation Database (gnomAD v3: gnomad.broadinstitute.org, covering 9 geographical areas), (ii) Exome Sequencing Project (ESP: evs.gs.washington.edu/EVS/), and (iii) 1000 Genomes Project (1KGP, www.internationalgenome.org).

## Abbreviations

1KGP: 1000 Genomes Project
ACE2: Angiotensin-converting enzyme 2
CoV: Coronavirus
COVID-19: Coronavirus Disease 2019
eQTL: Expression quantitative trait loci
gnomAD: Genome Aggregation Database
MERS: Middle East respiratory syndrome
SARS: Severe acute respiratory syndrome
SARS-CoV-2: Severe acute respiratory syndrome coronavirus 2
ESP: Exome Sequencing Project
S: Spike
TMPRSS2: Transmembrane serine protease 2
